# Cardiovascular Risk in Patients With Takayasu Arteritis Directly Correlates With Diastolic Dysfunction and Inflammatory Cell Infiltration in the Vessel Wall: A Clinical, *ex vivo* and *in vitro* Analysis

**DOI:** 10.3389/fmed.2022.863150

**Published:** 2022-05-16

**Authors:** Sebastiano Cicco, Vanessa Desantis, Antonio Vacca, Gerardo Cazzato, Antonio G. Solimando, Anna Cirulli, Silvia Noviello, Cecilia Susca, Marcella Prete, Gabriele Brosolo, Cristiana Catena, Aurelia Lamanuzzi, Ilaria Saltarella, Maria Antonia Frassanito, Antonella Cimmino, Giuseppe Ingravallo, Leonardo Resta, Roberto Ria, Monica Montagnani

**Affiliations:** ^1^Department of Biomedical Sciences and Human Oncology (DIMO), Unit of Internal Medicine and Clinical Oncology, University of Bari Aldo Moro Medical School, Bari, Italy; ^2^Department of Biomedical Sciences and Human Oncology, Pharmacology Section, University of Bari Aldo Moro Medical School, Bari, Italy; ^3^Division of Internal Medicine, Department of Medicine, University of Udine, Udine, Italy; ^4^Section of Pathology, Department of Emergency and Organ Transplantation, University of Bari Aldo Moro, Bari, Italy; ^5^Department of Admission and Emergency Medicine and Surgery, “S. Maria degli Angeli” Hospital, Azienda Sanitaria Locale (ASL) Bari, Bari, Italy; ^6^Department of Biomedical Sciences and Human Oncology (DIMO), General Pathology Unit, University of Bari Aldo Moro Medical School, Bari, Italy

**Keywords:** Takayasu arteritis (TAK), echocardiography, immune cell infiltration, vascular stiffness, T helper-like cells, Regulatory T Lymphocytes (Tregs)

## Abstract

**Background:**

Takayasu Arteritis (TAK) increases vascular stiffness and arterial resistance. Atherosclerosis leads to similar changes. We investigated possible differences in cardiovascular remodeling between these diseases and whether the differences are correlated with immune cell expression.

**Methods:**

Patients with active TAK arteritis were compared with age- and sex-matched atherosclerotic patients (Controls). In a subpopulation of TAK patients, Treg/Th17 cells were measured before (T0) and after 18 months (T18) of infliximab treatment. Echocardiogram, supraaortic Doppler ultrasound, and lymphocytogram were performed in all patients. Histological and immunohistochemical changes of the vessel wall were evaluated as well.

**Results:**

TAK patients have increased aortic valve dysfunction and diastolic dysfunction. The degree of dysfunction appears associated with uric acid levels. A significant increase in aortic stiffness was also observed and associated with levels of peripheral T lymphocytes. CD3^+^ CD4^+^ cell infiltrates were detected in the vessel wall samples of TAK patients, whose mean percentage of Tregs was lower than Controls at T0, but increased significantly at T18. Opposite behavior was observed for Th17 cells. Finally, TAK patients were found to have an increased risk of atherosclerotic cardiovascular disease (ASCVD).

**Conclusion:**

Our data suggest that different pathogenic mechanisms underlie vessel damage, including atherosclerosis, in TAK patients compared with Controls. The increased risk of ASCVD in TAK patients correlates directly with the degree of inflammatory cell infiltration in the vessel wall. Infliximab restores the normal frequency of Tregs/Th17 in TAK patients and allows a possible reduction of steroids and immunosuppressants.

## Introduction

Vasculitis of large vessels is an inflammation of the wall of large and medium-sized arteries, including the aorta and its main branches and the pulmonary artery (1). The clinical signs and symptoms are due to both systemic inflammation and local vascular complications and are associated with elevated inflammatory markers (1). Among these diseases, Takayasu arteritis (TAK) is a chronic granulomatous vasculitis. The majority of TAK patients present with concurrent nonspecific inflammatory signs and symptoms, primarily associated with local stenosis (93%), occlusion (57%), dilatation (16%), and aneurysm formation (7%) (2).

Abnormal immune response is a crucial factor in the pathogenesis of TAK. Regulatory T lymphocytes (Tregs), a subset of CD4^+^ T cells that express high levels of both CD25 (the α-chain of the high-affinity interleukin-2 receptor) and the transcription factor forkhead box protein P3 (Foxp3), are central mediators of peripheral tolerance (3). Under certain conditions, Tregs can differentiate into T helper (Th)-like cells, leading to a drastic change in their immune functions. Indeed, recent evidence suggests that Tregs can differentiate into Th1, Th2, or Th17 cells, leading to a shift from an immunosuppressive function to a role in the pathogenesis of autoimmune diseases (4). The potential role of Tregs and their associated cytokine secretion in TAK patients is under active investigation to expand the horizon for more effective therapies.

Because of the inflammatory nature of TAK, first-line treatment generally consists of high-dose steroids (usually prednisone). Commonly prescribed second-line agents include immunosuppressants (2). Although these traditional agents can be effective in inducing remission of TAK, relapses remain common when prednisone is discontinued (5, 6). Recent advances in the treatment of TAK patients have been based on the increased understanding of the pathophysiology of TAK and the concurrent availability of new biologic therapies (7). To date, the administration of anti-tumor necrosis factor-alpha (TNF-α) agents, such as infliximab, appears to be a valuable and safe alternative to standard therapy (4). By blocking TNF-α-induced activation of inflammatory signals, infliximab leads to long-term clinical improvement with significant benefits for patients' quality of life (8, 9).

TAK patients are likely to have lesions at different vascular levels, mostly at the subclavian and carotid levels, with either bilateral involvement or focal involvement confined to one carotid or subclavian artery, which can be monitored by ultrasonography (10, 11). At the onset, pathophysiological features are usually nonspecific and include systemic disturbances such as fever, anorexia, weight loss, night sweats, and fatigue. As the disease progresses, diffuse proliferation of the arterial tunica intima along with fibrotic and stenotic lesions may cause ischemic symptoms, whose clinical manifestations depend on the location of the affected arteries. Further progression of TAK leads to destruction of the tunica media, which is often accompanied by aortic regurgitation as well as aneurysm and vessel rupture (7).

Few data are available on ultrasound-based assessment of cardiac function in large vessel vasculitis, although features of early cardiac involvement have been described in other autoimmune diseases (12, 13). Nevertheless, an increase in left ventricular mass (14) and indirect involvement of the right ventricle, secondary to pulmonary hypertension, have been detected by echocardiography in TAK patients (15). Thus, as with several other diseases associated with inflammatory features in the arterial wall, such as hypertension and atherosclerosis (16–18), changes in cardiac structure and function may characterize the progression of TAK disease, increasing the overall risk of cardiovascular morbidity/mortality (19, 20). However, ultrasonographic parameters that can accurately characterize cardiovascular risk and correlations between inflammatory parameters and cardiovascular structural changes have not been studied in TAK patients.

Cardiovascular risk data are based on the assessment of vascular changes (21). The increased vascular resistance is the consequence of many phenomena associated with the development of atherosclerosis (21). The cornerstone of this evolution is inflammation, which is due to increased infiltration of immune cells into the vessel wall (21–23). In classic cardiovascular diseases, there are accepted parameters for assessing organ damage, which represent the referring values also in ultrasound assessment (24). The aim of this study was to compare vascular and cardiac ultrasonography parameters predictive of increased cardiovascular risk in TAK patients, with respect to values obtained in hypertensive and atherosclerotic patients. We also investigated the relationship between cardiac and carotid artery changes (by ultrasonography) and peripheral blood inflammatory cell concentrations. Finally, we investigated the potential role of biomarkers (frequency of Treg and Th17 cells) in TAK-refractory patients, and found that treatment with infliximab ameliorated the Tregs and Th17 ratio with a concomitant clinical improvement. Overall, the study of Tregs and Th17 populations might represent an additional, novel therapeutic approach to identify cardiovascular risk in TAK patients.

## Materials and Methods

### Patients and Study Design

This cross-sectional single-center study was conducted in conformity to the Good Clinical Practice Guidelines of the Italian Ministry of Health and the ethical guidelines of the Declaration of Helsinki (as revised and amended in 2004) and with the approval of the Ethics Committee of the University of Bari Medical School (Code number 06R76Y9-1).

Thirty TAK patients (22 female, 8 male, aged 49 ± 14 years) were consecutively enrolled and followed-up from January 2014 to December 2018 among patients with large vessel vasculitis admitted to our Center. Patients met the European League against Rheumatism criteria for large vessel vasculitides (25). Patients were in the active phase of the disease (ITAS-A > 6) and were clinically assessed before (time point T0) and 18 months after (T18) treatment with steroids and infliximab (5 mg/kg) therapy (26). However, steroids were tapered within 5-6 months and generally stopped. The Control group consisted of 30 age- and sex-matched patients (22 F, 8 M, 51 ± 12 years old) who received an ultrasound diagnosis of atherosclerosis according to ESC guidelines (24, 27) and had no history of chronic viral infection, autoimmune disease, immune-mediated disease, hematologic malignancies, or cancer. All patients underwent a comprehensive medical examination, including medical history (age, sex, smoking habits, drug treatment, and concomitant diseases) and physical examination.

### Clinical and Laboratory Evaluation

Blood pressure values were measured with an electronic sphygmomanometer and were reported as the mean of three consecutive in-office measurements in the supine position after 15 min of rest. The 10-year estimation of the risk of cardiovascular events was performed for all patients using the ACC/AHA validated ASCVD score (28).

At patients admission, levels of erythrocyte sedimentation rate (ESR), creatinine, blood glucose, total cholesterol, high-density lipoprotein (HDL), low-density lipoprotein (LDL), triglycerides, uric acid, CRP, and C3 complement fraction were measured by commercial laboratory diagnostic kits. A standard lymphocytogram was performed in 10 TAK and 8 control patients.

### Ultrasound and Imaging Evaluation

A standardized method included a transthoracic echocardiogram with Doppler evaluation (ETG) and ultrasonography Doppler evaluation (SAD) of the supra-aortic vessels (29). All ultrasound measurements were performed with a 2.5-MHz probe (MyLabSeven Doppler echocardiography device, Esaote, Italy) in the left lateral decubitus position; SAD was performed with a 12-MHz linear probe (MyLabSeven Doppler device, Esaote, Italy) on the relaxed neck in the supine position.

#### Echocardiogram and Cardiac Doppler

Using a previously standardized flow-chart (30), two-dimensional echocardiography was performed to measure aortic root diameter (Ao) in parasternal long-axis view during both systolic (AoS) and diastolic phases (AoD) by an ECG-guided point measurement. In the same position, one-dimensional echocardiography (M-mode) was used to obtain end-diastolic measurements of interventricular septum thickness (IVS), posterior wall thickness (PWD), and left ventricular internal diameter (LVD). According to the international guidelines, the left ventricular mass (LVM) was calculated using the Devereux formula for M-mode diameters. Relative wall thickness (RWT) was calculated using the international validated formula (31). An apical four-chamber view (A4C) was used to measure tricuspid annular plane systolic excursion (TAPSE) and right atrial area (RA). The A4C view was used to measure left ventricular end-diastolic volume (LVEDV), ejection fraction (EF), and left atrial volume (LAV). Sub-costal view (SC) was used to measure inferior vena cava diameter (CVD). Ao, LAV and LVM were indexed to body surface area (BSA) and height 2.7 (LVMi2.7) (24). Aortic root stiffness was assessed using the Aortic Stiffness Index (ASI = In (SBP/DBP)/[(AoS – AoD)/AoD] (32). Doppler measurements were performed in the A4C B-mode view. The velocity of tricuspid regurgitation (TRV) was determined using a continuous wave Doppler curve of tricuspid regurgitation (TR) trace. The peak value of TRV was used to measure the pressure difference between the right ventricle and right atrium (RA) according to the simplified Bernoulli equation (*P* = 4[TRmax]2). RA filling pressure was estimated from the diameter and respirophasic variability of the inferior vena cava during normal breathing. The pulmonary arterial pressure (PAP) estimate was a derived sum of RA filling pressure and TR pressure. In the same view, pulsed-wave Doppler was used to measure transmitral velocity E and A and septal velocity e' in tissue Doppler imaging (TDI) mode. Diastolic dysfunction was assessed as the combination of mitral ratio E/A and E/e' and were stratified as indicated in guidelines (31).

#### Carotid Ultrasound Doppler

This evaluation was performed to assess vascular damage. Extensive evaluation was performed in both groups, looking to carotid, upper and lower limbs arteries, as well as aorta, kidney, and splanchnic arteries. In TAK patients, the vessel wall dysregulated activity was investigated by evaluation of vascular dilation as well as vessel wall oedema or neovascularization using contrast-enhancement Doppler ultrasound. Carotid intima-media thickness (IMT) was considered as standard measure between the two groups and was sampled using basal B-mode imaging. Selecting the best view of the common carotid artery, radiofrequency analysis yields the mean IMT based on ten automated measurements taken on the posterior wall of the common carotid artery at a length of 1 and 1 cm distal to the vessel bifurcation. Blood flow was analyzed with PW Doppler at a standard angle of 60°, measuring the flow of the common and internal carotid arteries. The North American Symptomatic Carotid Endarterectomy Trial (NASCET) method was used to quantify arterial stenosis and atherosclerosis (33).

#### Advanced Imaging Technique

After the evaluation of ultrasound, Computerized Tomography angiography was performed in TAK patients. On the contrary, Controls underwent only ultrasound vascular evaluation according to ESC guidelines (27) and CT scan was performed only in critical vascular damage. Therefore, these data were not used in comparison.

### Histological Analysis

Ten TAK patients and eight Controls underwent surgery for critical supraaortic vessel stenosis resolution. In these patients, surgical samples were collected to evaluate immune cell infiltration. Samples were fixed in neutral 10% buffered formalin, dehydrated and enclosed in paraffin. Five micrometer thick slices were taken from the paraffin-embedded blocks, deparaffinized, rehydrated and routinely stained with hematoxylin-eosin. Immunohistochemistry was then performed using antibodies for the following markers: monoclonal mouse anti-human CD4 (Agilent, DAKO Omnis, Carpinteria, CA, USA, Cat. M7310), monoclonal mouse anti-human CD8 (Novacastra Laboratories Ltd., Cat. NCL-L-CD8-4B11), polyclonal rabbit anti-human CD3 (Agilent, DAKO Omnis, Cat. GA503) and monoclonal mouse anti-human CD15 (Agilent, DAKO Omnis, Cat. GA062).

To study the infiltration of immune cells, the expression of HMGB1 (34) was assessed using polyclonal rabbit anti-HMGB1 serum (Ab18256, Abcam, Cambridge, USA). Blocks were pretreated on PT-LINK (DAKO) instrument with EDTA [EnVision Flex, Target Retrieval Solution, High Ph (50x), DAKO] for CD3, CD4, CD8 antibodies and Citrate [EnVision Flex, Target Retrieval Solution, Low Ph (50x), DAKO] for HMGB1 antibodies. Immunohistochemistry was performed to measure the density of CD4^+^ and CD8^+^ cells in 10 fields at 400× magnification of each sample. One field measured 140 micrometers in length and 110 micrometers in width, and the total amplitude was 15,400 micrometers squared. A Reichert Polyvar 2 microscope with a JTV digital telecamera and a Trinitron monitor (Sony) was used. HMGB1 expression was assessed by highlighting the chromogen signal on the plasma membrane, nucleus, cytoplasm or extracellular medium of the samples examined. The relative expression level was calculated by adding the degree of staining intensity (grade 0 = no staining; grade 1 = weak staining; grade 2 = moderate staining; grade 3 = intense staining) with the percentage of mass extension (score 0: <1%; score 1: 1-25%; score 2: 26-50%; value 3: 51-74%; score 4: ≥75%). The resulting final scores were rated as high (if > 3) or low (if ≤ 3). After processing, two different expert pathologists scored the samples. The final value reported represents the mean of the two values.

### Biological Samples and Cell Preparations

Sera were obtained after centrifugation of clotted blood samples and stored at −20°C until further analysis. PBMCs were isolated from heparinized samples using Ficoll-Hypaque (GE Healthcare Life Sciences) gradient separation. CD4^+^CD25^+^CD127^−/dim^ T cells were purified using the CD4^+^CD25^+^CD127^−/dim^ regulatory T Cell Isolation Kit II (Miltenyi Biotec, Auburn, CA, USA). The obtained cell populations had a purity of 95% as shown by flow cytometry on immunostained cells.

### Cell Cultures and Stimulation

PBMCs (6 x 10^5^/well) were cultured in triplicate in 96-well round-bottom plates in 200 ml of Roswell Park Memorial Institute (RPMI) 1640 medium supplemented with 10% of heat-inactivated FBS, 2 mM of L-glutamine, 100 U/ml of penicillin, and 100 mg/ml of streptomycin (all from Sigma-Aldrich, St Louis, MN, USA). Cells were untreated and/or treated with 10 ng/ml of PMA and 1 mg/ml of ionomycin (all from Sigma-Aldrich) for 18 h in a humidified atmosphere containing 5% CO2. After 5 h, 3μM of monensin was added to block Golgi transport (Sigma-Aldrich). Cells were then harvested and immunostained.

### Cytofluorimetric Staining

A set of commercial monoclonal antibodies (mAbs) have been used in flow cytometry to analyze the expression of Tregs. Peridinin-chlorophyll proteins cyanine 5.5 conjugated (Perce) anti-CD4 mAb, phycoerythrinCyanine7 conjugated (PECy7) anti-IL-17 mAb, and the alloficocianin- conjugated (APC) anti-Foxp3 mAb were all part of the Human Th17/Treg phenotyping kit (Cat. 560762, Becton Dickinson-BD Biosciences, San Jose, CA, USA). Fluorescein isothiocyanate (FITC) conjugated anti-CD4 mAb and FITC conjugated anti-CD3 mAb were purchased from Beckman Coulter (Brea, California, USA) (Cat. 345768 and Cat. 557851, respectively). Cells were incubated with mAbs to surface antigens for 30 min at 4°C and then washed twice in cold phosphate-buffered saline (PBS) (Sigma-Aldrich) containing 0.1% fetal bovine serum (FBS) (Sigma-Aldrich) before flow cytometry was performed. To determine Th17/Treg phenotype, cells were fixed, permeabilized, and stained according to the manufacturer's instructions for the Human Th17/Treg Phenotyping Kit (BD Biosciences). Stained cells were acquired using FACSCanto II cytofluorimetry (BD Biosciences) and analyzed using FACSDiva software (BD Biosciences).

### Statistics

Data were analyzed using GraphPad Prism software (La Jolla, CA, USA) and expressed as means ± S.D. Chi-square test was performed to analyze the distribution of dichotomous values. The non-parametric Mann-Whitney test for comparisons and Spearman distribution for correlations were performed for non-normally distributed data. The parametric unpaired *t*-test for comparisons and Pearson distribution for correlations were performed for normally distributed data. A value of *p* < 0.05 was taken as an indication of statistical significance.

## Results

### Baseline Features and Clinical Differences

Baseline history information and clinical parameters for all patients are summarized in Tables 1, 2. While no significant difference was found in the incidence of overweight/obesity (Table 1), body mass index (BMI) (25.21 ± 5.21/28.44 ± 4.22) and body surface area (BSA) (1.73 ± 0.29/1.89 ± 0.23) values tended to be lower in TAK patients than in control group. Total cholesterol (171.04 ± 43.66/192.01 ± 32.83) and LDL cholesterol (96.36 ± 34.03/113.82 ± 29.34) were slightly albeit significantly lower in the TAK group, while the values for HDL (55.96 ± 23.39/56.63 ± 11.24) or triglycerides (103.90 ± 37.95/107.91 ± 42.56) overlapped between the groups (Table 2). Accordingly, the estimated incidence of atherosclerosis was low in our TAK patients and treatment with statins was less frequent than in the control patients (Table 1).

**Table 1:** Baseline history information of enrolled patients.

**Characteristic**	**Takayasu**	**Controls**	***P*-value**
	**arteritis**		
Age (years)	49.27 ± 18.87	51.43 ± 12.51	Ns
F/M	22/8	22/8	Ns
BMI (kg/m^2^)	25.59 ± 5.7021	28.44 ± 4.22	Ns#
BSA (m^2^)	1.73 ± 0.31	1.89 ± 0.23	Ns#
Overweight/obesity (*n*°)	9/5	12/7	Ns
Arterial hypertension (*n*°)	12	30	0.001
Diabetes (*n*°)	3	4	Ns
Ischemic heart disease (*n*°)	4	3	Ns
Ischemic brain disease (*n*°)	3	2	Ns
Preserved EF heart failure (*n*°)	3	5	Ns
Smoke (*n*°)	5	7	Ns
Cigarettes (*n*/day) [IQR]	15 [7.5-18]	13 [7-20]	Ns
ASCVD (%)	21.69 ± 16.09	8.55 ± 7.63	0.001#
**Medications**			
Anti-hypertensives drugs (*n*°)	12	30	0.001
Antiplatelet (*n*°)	11	10	Ns
Statins (*n*°)	3	25	0.001
Oral antidiabetic drugs (*n*°)	3	3	Ns
Insulin (*n*°)	1	1	Ns
Steroids (*n*°)	22	-	
Prednisone equivalent (mg/day)	27.36 ± 17.86	-	
DMARDs (*n*°)	22	-	
Infliximab (*n*°)	16	-	

*ASCVD, atherosclerotic cardiovascular disease; BMI, body mass index; BSA, body surface area; DMARDS, disease modified anti rheumatic drugs; EF, ejection fraction. Ns, not significant. Data were analyzed using Chi-Squared test or Student T-test (#)*.

**Table 2:** Clinical and routine parameters of enrolled patients.

**Characteristic**	**Takayasu**	**Controls**	***P*-value**
	**arteritis**		
SBP (mmHg) right	125.30 ± 20.34	123.35 ± 10.27	Ns
SBP (mmHg) left	120.65 ± 30.31	125.11 ± 9.32	Ns
DBP (mmHg) right	70.71 ± 2.495	78.93 ± 6.10	0.0004
DBP (mmHg) left	72.35 ± 8.00	79.73 ± 5.01	0.0002
HR (bpm)	76.28 ± 13.99	67.80 ± 8.57	0.0076
Creatinine (mg/dl)	0.92 ± 0.44	0.81 ± 0.21	Ns
Creatinine Clearance (ml/min)	89.93 ± 34.32	87.17 ± 16.43	Ns
Glycaemia (mg/dl)	98.82 ± 32.16	91.47 ± 14.26	Ns
Total Cholesterol (mg/dl)	171.04 ± 43.66	192.01 ± 32.83	0.042
HDL (mg/dl)	55.96 ± 23.39	56.63 ± 11.24	Ns
LDL (mg/dl)	96.36 ± 34.03	113.82 ± 29.34	0.041
Triglyceride (mg/dl)	103.90 ± 37.95	107.91 ± 42.56	Ns
Uric Acid (mg/dl)	4.30 ± 1.98	4.65 ± 1.19	Ns

*SBP, systolic blood pressure; DBP, diastolic blood pressure; HR, heart rate; HDL, high-density lipoprotein; LDL, low-density lipoprotein. Ns, not significant*.

Although the use of antihypertensive medications was less frequent in TAK patients (Table 1), no difference was observed in systolic blood pressure (SBP) values, either on the right arm (125.30 ± 20.3/4123.35 ± 10.27) or on the left arm (120.65 ± 30.31/125.11 ± 9.32) (Table 2). Instead, diastolic blood pressure (DBP) was significantly lower in both the right (70.71 ± 2.495/78.93 ± 6.10) and left arms (72.35 ± 8.00/79.73 ± 5.01) in TAK patients than in Controls (Table 2). Heart rate (HR) was significantly higher in TAK patients (76.28 ± 13.99/67.80 ± 8.57) than Controls (Table 2). Thus, there was no difference in individual cardiovascular risk factors between the two groups of enrolled patients.

### Ultrasound Evaluation and Vascular Damage

Interventricular septum (IVS) (11.88 ± 2.03/11.88 ± 1.64) and left atrial volume (LAV) (65.16 ± 32.44/63.57 ± 16.21) were increased in patients with TAK, overlapping with values in Controls (Table 3). Also, left ventricular mass (LVM) (205.30 ± 63.11/205.91 ± 44.73), LVM indexed for BSA (LVMi) (118.70 ± 32.20/117.04 ± 21.89), and height2.7 (LVMi2.7) (56.07 ± 19.25/58.37 ± 12.01) were similar in both groups and always increased in Controls compared with the normal population (24, 25). Although most echocardiographic parameters were not significantly different between TAK and Controls (Table 3), TAK patients showed a trend to increase LAV indexed for BSA (LAVi) (37.42 ± 16.54/30.69 ± 16.54) (Table 3). These results suggest that, although similar cardiac remodeling was observed in both groups, TAK may have increased volume overload in the left heart.

**Table 3:** Echocardiographic parameters of enrolled patients.

**Parameter**	**Takayasu**	**Controls**	***P*-value**
	**arteritis**		
IVS (mm)	11.88 ± 2.03	11.88 ± 1.64	Ns
LvedD (mm)	47.02 ± 5.76	46.89 ± 4.48	Ns
PWT (mm)	11.38 ± 1.45	11.44 ± 1.25	Ns
LVedVol	87.24 ± 26.70	77.69 ± 15.62	Ns
LVM (gr)	205.30 ± 63.11	205.91 ± 44.73	Ns
LVMi (gr/m^2^)	118.70 ± 32.20	117.04 ± 21.89	Ns
LVMi2.7	56.07 ± 19.25	58.37 ± 12.01	Ns
Aod (mm)	32.71 ± 4.56	31.57 ± 3.36	Ns
Aoi (mm/m^2^)	19.26 ± 2.49	17.24 ± 2.15	Ns
RWT	0.48 ± 0.08	0.49 ± 0.07	Ns
LAV (ml)	65.16 ± 32.44	63.57 ± 16.21	Ns
LAVi (ml/m^2^)	37.42 ± 16.54	30.69 ± 16.54	0.01
Ejection fraction (%)	61.42 ± 5.41	61.83 ± 2.73	Ns
E velocity (cm/s)	69.44 ± 16.45	56.43 ± 15.42	Ns
A velocity (cm/s)	65.63 ± 26.40	67.07 ± 15.63	Ns
e' velocity (cm/s)	8.20 ± 0.51	7.23 ± 2.41	Ns
E/e' ratio	10.82 ± 7.32	9.10 ± 2.89	Ns
IMT (mm)	1.93 ± 0.79	1.66 ± 1.32	Ns
Aortic wall thickness (mm)	3.96 ± 0.80	2.83 ± 0.57	0.001
Diastolic dysfunction (none/I/II/severe)	9/10/9/2	6/19/5/0	0.049#
Aortic regurgitation severity	17/8/5	26/3/1	0.033#
(none/mild-to-moderate/			
moderate-to-severe)			

*IVS, interventricular septum; LvedD, left ventricle end-diastolic diameter; PWT, posterior wall thickness; LVM, left ventricle mass; LVMi, LVM indexed for BSA; Aod, aortic diameted; RWT, relative wall thickness; LAV, left atrial volume; LAVi, LAV indexed for BSA; IMT, intima-media thickness. Ns, not significant. Data were analyzed using Student T-test or Chi-Squared test (#)*.

**Figure 1:**
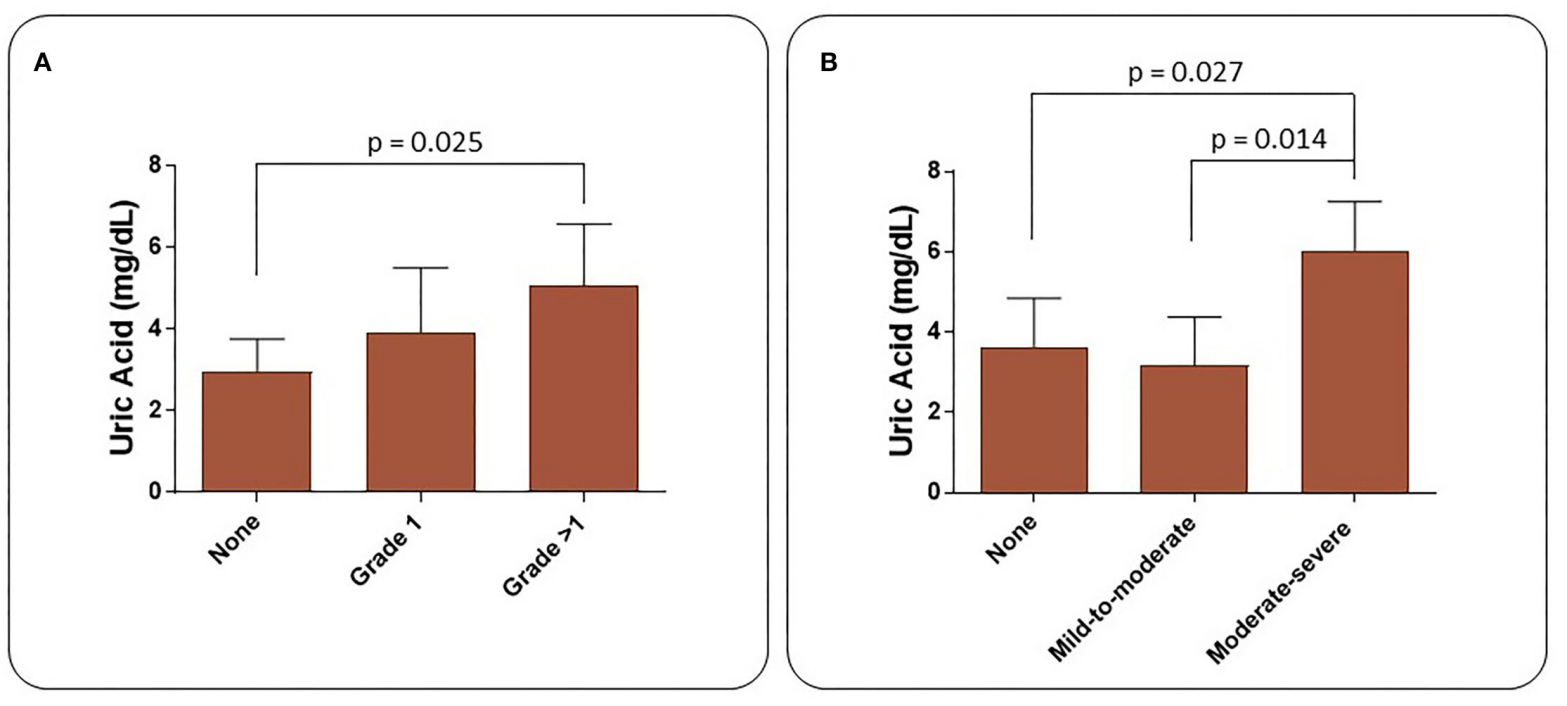
Uric acid levels in TAK patients increase according to the severity of heart diastolic dysfunction **(A)** and aortic valve regurgitation **(B)**.

**Figure 2:**
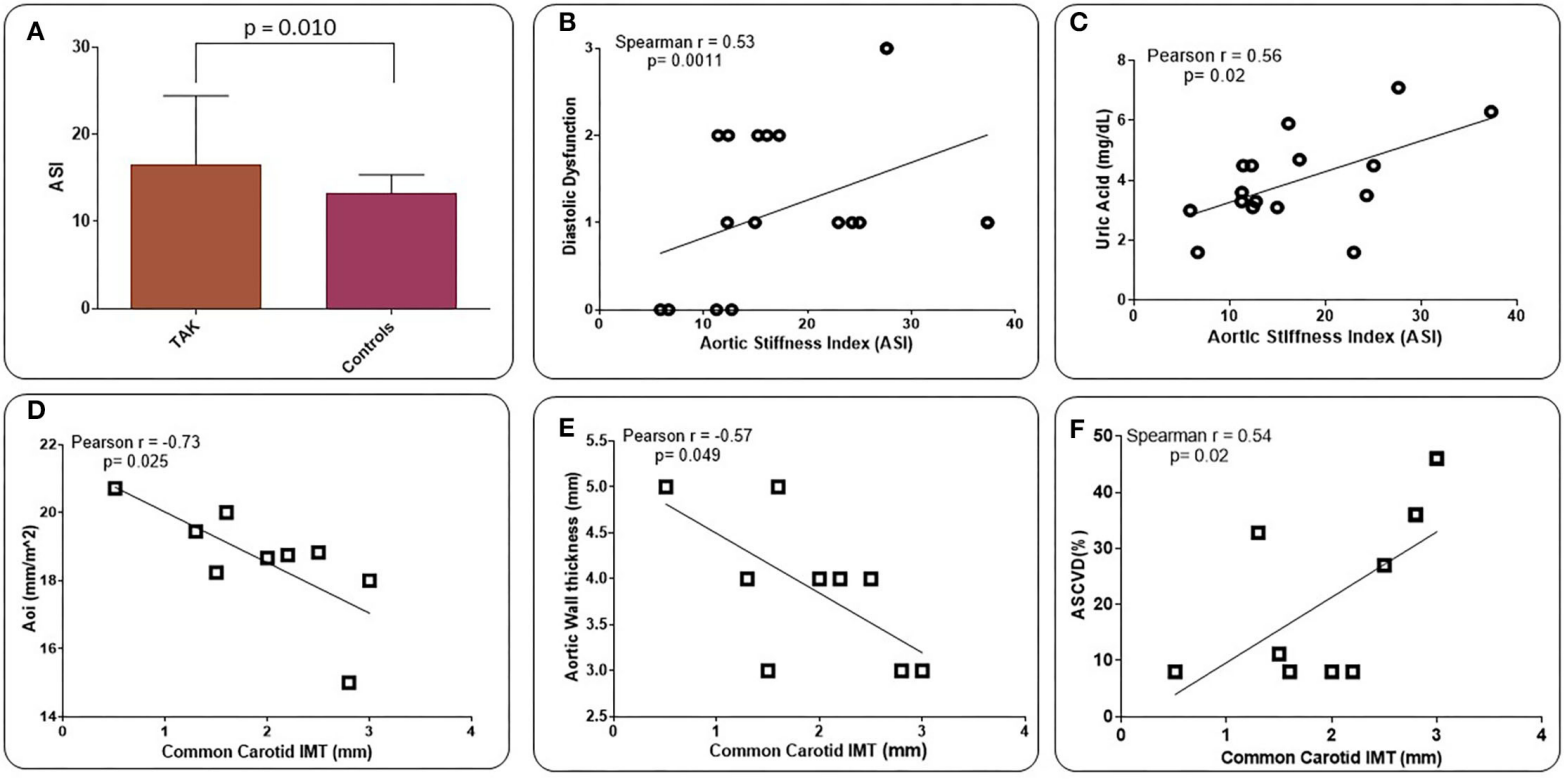
Values of aortic vascular stiffness (ASI) in TAK patients compared with Controls (atherosclerotic patients) **(A)**. ASI values are directly related to the degree of LV diastolic dysfunction **(B)** and uric acid (UA) levels **(C)** in TAK patients. Common carotid intima-media thickness (IMT) shows an inverse correlation with aortic diameter indexed by body surface area (BSA) (Aoi) **(D)** and aortic wall thickness **(E)**, and a direct correlation with ASCVD 10-year risk rate **(F)** in TAK patients. Evaluation was performed excluding already known cardiovascular disease **(B,C)** and those who underwent to surgical treatment **(D-F)**.

Increased diastolic dysfunction and aortic regurgitation were also observed in TAK patients (Table 3). Consistent with the evidence that diastolic dysfunction is associated with increased uric acid levels (35), a marker of endothelial stress, we found that uric acid levels were significantly elevated in TAK patients with severe aortic regurgitation compared with TAK patients without dysfunction (5.06 ± 1.51/2.96 ± 0.79). Accordingly, TAK patients with moderate-severe aortic regurgitation had elevated uric acid levels compared with patients without regurgitation (6.03 ± 1.22/ 3.64 ± 1.21) or mild-to-moderate diastolic dysfunction (6.03 ± 1.22/3.18 ± 1.20) (Figure 1A). Although uric acid levels were not significantly different between TAK and patients (4.30 ± 1.98/4.65 ± 1.19) (Table 2), a correlation was observed between the severity of diastolic dysfunction and elevated uric acid levels in patients in the TAK group (Figure 1B). On the same line, an increase in arterial stiffness (ASI) (16.54 ± 7.88/13.28 ± 3.11) was observed in TAK patients compared with Controls (Figure 2A). Moreover, TAK patients without an already known cardiovascular disease showed a significant positive correlation with diastolic dysfunction (Figure 2B) and with uric acid levels (Figure 2C). This suggests that TAK patients have an increased vessel remodeling.

A statistically significant increase in aortic wall thickness was measured in TAK patients compared with Controls (1.93 ± 0.79/1.66 ± 1.32) (Table 3). Standard carotid intima-media thickness (IMT) showed no difference between patients of both groups. Different branches involved into the two groups and ultrasound activity patterns are listed in Supplementary Table 1. Active wall remodeling in TAK patients was described as 12.5% vascular dilation, 46.7% of wall neovascularization, and 66.7% oedema. No differences were found in vascular involvement between the two groups (Supplementary Table 1). However, in those TAK patients who did not undergo surgical treatment, the thickness of the common carotid wall was inversely related to the aortic diameter when indexed with the BSA (Aoi), as well as to the aortic wall thickness (Figures 2D,E). Interestingly, IMT of the common carotid artery was significantly correlated with atherosclerotic cardiovascular disease (ASCVD) in TAK (Figure 2F) but not in controls. These data suggest that cardiovascular risk in TAK patients is promoted by enhanced vascular remodeling rather than by classic risk factors.

### Immune Cells and Vascular Involvement

A significant decrease in peripheral blood lymphocyte count was observed in TAK patients compared to Controls (1584.4 ± 554.6/2178.6 ± 626.5, Table 4). No other significant differences were observed between the two groups. As expected, ESR (41.48 ± 31.49/10.15 ± 6.79) and C-reactive protein (CRP) values (46.30 ± 57.44/3.01 ± 0.45) were significantly higher in TAK patients than in Controls (Table 4).

**Table 4:** Peripheral blood immunological parameters of enrolled patients.

**Characteristic**	**TAK**	**Controls**	***P*-value**
Total WBC count (cell/ml)	7,226.3 ± 1,823.4	8337.8 ± 3274.6	Ns
Lymphocytes (cell/ml)	1,584.4 ± 554.6	2,178.6 ± 626.5	0.01
CD3^+^ (cell/ml)	1,331.1 ± 504.0	1,864.2 ± 784.6	Ns
CD3^+^ HLA-DR^+^ (%)	3.75 ± 3.59	3.50 ± 2.08	Ns
CD3^+^ CD4^+^ (cell/ml)	738.8 ± 301.8	1,064.3 ± 544.5	Ns
CD3^+^ CD8^+^ (cell/ml)	560.3 ± 250	719.0 ± 325.5	Ns
CD3^+^ CD16/56^+^ (cell/ml)	135.7 ± 98.86	226.9 ± 167.0	Ns
CD19^+^ (cell/ml)	123.0 ± 159.7	156.7 ± 104.9	Ns
Neutrophils (cell/ml)	4,902.2 ± 2193.4	5,536.2 ± 2702.2	Ns
NLR	4.43 ± 4.17	2.94 ± 2.07	Ns
ESR (mm/h)	41.48 ± 31.49	10.15 ± 6.79	0.0001
CRP (mg/dl)	46.30 ± 57.44	3.01 ± 0.45	0.0001
C3 (g/L)	1.19 ± 0.26	1.24 ± 0.28	Ns

*WBC, white blood cells; NLR, neutrophil to lymphocyte ratio; ESR, erythrocyte sedimentation rate; CRP, C-reactive protein; C3, complement component 3. Ns, not significant*.

Based on the immunological analysis, we found that ASI values were negatively correlated with peripheral total white blood cell counts (WBCs), neutrophils and CD3^+^CD8^+^, CD3^+^HLA-DR^+^ counts, and neutrophil-to-lymphocyte ratio (NLR). Conversely, ASI values showed a significant positive correlation with total lymphocyte count and total CD3^+^ and CD3^+^CD4^+^ cell count (Table 5).

**Table 5:** Pearson correlation analysis between Aortic Stiffness Index and peripheral blood immunological parameters and blood pressure of Takayasu patients.

**Characteristic**	**R**	***P*-value**
Total WBC count	−0.85	0.007
Lymphocytes	0.91	0.002
CD3^+^	0.97	0.001
CD3^+^ HLA^−^DR^+^	−0.98	0.0005
CD3^+^ CD4^+^	0.75	0.03
CD3^+^ CD8^+^	−0.84	0.008
CD3^+^ CD16/56^+^	0.16	Ns
CD19^+^	0.32	Ns
Neutrophils	−0.93	0.001
NLR	−0.97	0.001
ESR	0.21	Ns
CRP	0.02	Ns
Uric Acid	0.57	0.04
SBP	0.68	0.03

*WBC, white blood cells; NLR, neutrophil-lymphocyte ratio; ESR, erythrocyte sedimentation rate; CRP, C-reactive protein; SBP, systolic blood pressure. Ns, not significant*.

A positive significant correlation was found for aortic wall thickness with CD3^+^HLA-DR^+^ and CD3^+^CD4^+^ peripheral cells, while this vessel parameter had a significant negative correlation with the total number of CD3^+^, CD3^+^CD8^+^ and NLR (Table 6). Finally, SBP levels were significantly correlated with ASI (Table 5) but negatively correlated with aortic wall thickness (Table 6).

**Table 6:** Pearson correlation analysis between Aortic wall thickness and peripheral blood immunological parameters and blood pressure of Takayasu patients.

**Parameter**	**R**	***P*-value**
Total WBC count	0.009	Ns
Lymphocytes	−0.91	0.0007
CD3^+^	−0.79	0.005
CD3^+^ HLA^−^DR^+^	0.86	0.003
CD3^+^ CD4^+^	0.62	0.03
CD3^+^ CD8^+^	−0.87	0.003
CD3^+^ CD16/56^+^	−0.09	Ns
CD19^+^	−0.09	Ns
Neutrophils	0.27	Ns
NLR	−0.97	0.001
ESR	−0.44	Ns
CRP	−0.31	Ns
Uric Acid	−0.44	Ns
SBP	−0.55	0.049

*WBC, white blood cells; NLR, neutrophil-lymphocyte ratio; ESR, erythrocyte sedimentation rate; CRP, C-reactive protein; SBP, systolic blood pressure. Ns, not significant*.

Overall, these findings support the hypothesis that increased vascular inflammation in TAK patients is a major determinant for the reduced vascular compliance. No correlation was found to disease activity nor to different sites of disease (data not shown).

### Immune Cell Infiltration in TAK and Atherosclerotic Patients

To confirm the possible relationship between infiltration by immune cells and the extent of vascular lesions in TAK patients, *ex vivo* experiments were performed on blood vessel sections from patients of both groups.

**Figure 3:**
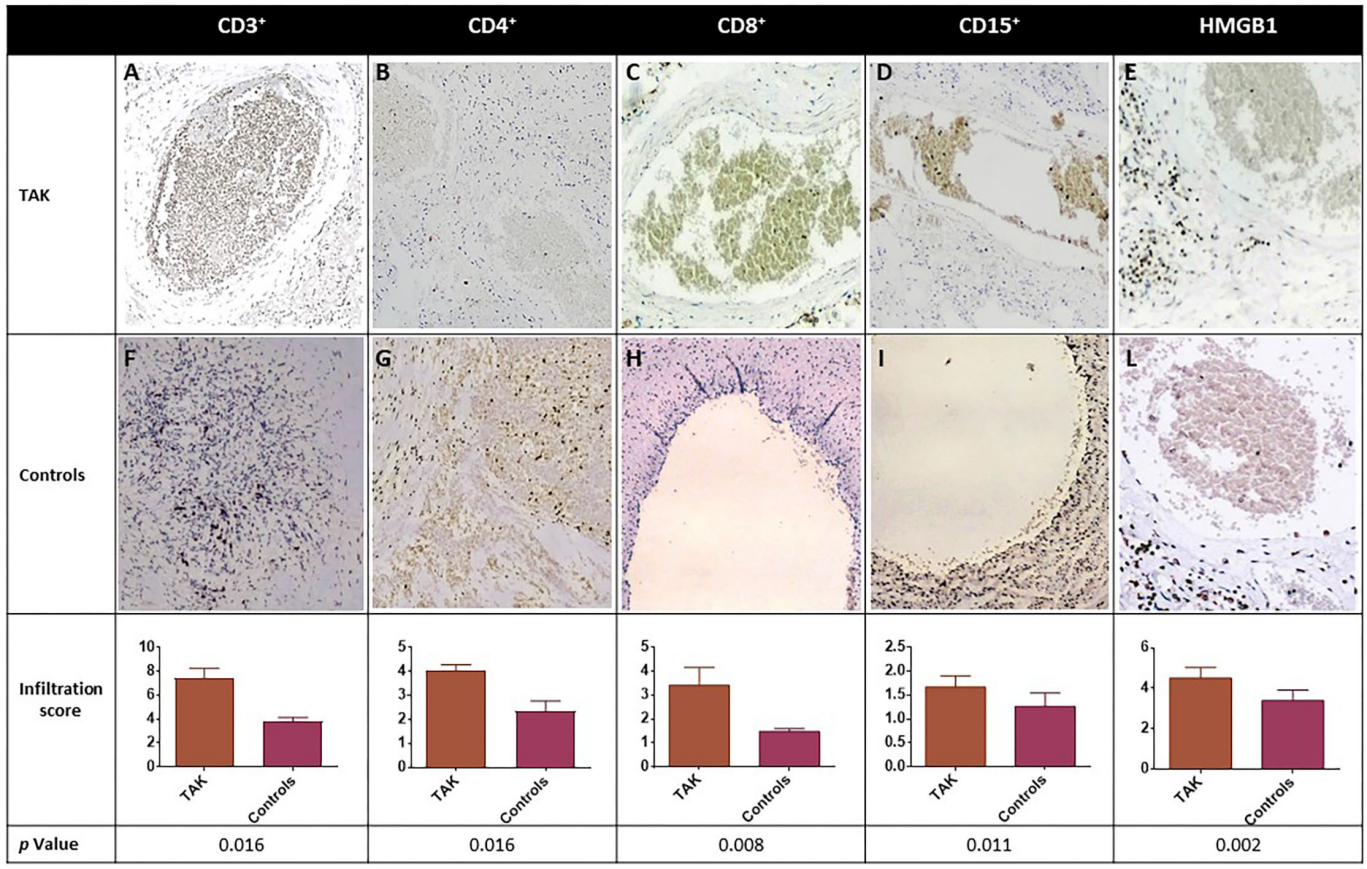
Immune cell infiltration (CD3^+^, CD4^+^, CD8^+^, and CD15^+^) and expression level of inflammation-associated cytokines of vascular injury HMGB1 in biopsy samples from blood vessels of TAK patients **(A-E)** and Controls **(F-L)**. Representative immunohistochemistry (original magnification: 20X) is shown in the top and middle panels. The lower panel shows the results as mean ± S.D. of three independent experiments for each field.

A significant increase in CD3^+^, CD4^+^, and CD8^+^ cell infiltration was observed in samples from TAK patients compared with Controls (Figure 3). High-mobility group box 1 (HMGB1) staining, which labels immunological inflammatory cells, was also significantly higher in the TAK group (Figure 3).

To evaluate neutrophilic vessel wall infiltration, CD15^+^ immune cells were stained and their level was found significantly higher in TAK-derived vessels than in samples from Controls (Figure 3). Moreover, in TAK vessels, there was a significant direct correlation between CD4^+^ (T helper or Th) cell infiltration and CD15^+^ cells (r = 0.800, *p* = 0.038), but not with the total number of CD3^+^ or CD8^+^ cells. Both CD15^+^ and CD4^+^ cells were directly correlated with HMGB1 (r = 0.894, *p* = 0.029) (Figure 3), suggesting an association with inflammation-related vascular damage. No significant histological correlation was observed in samples from atherosclerotic patients.

In line with previous observations, histology results support the concept that infiltration by immune cells is a key feature of vascular damage and chemotactic cytokines release.

### Frequency of Treg and Th17 Cells

**Figure 4:**
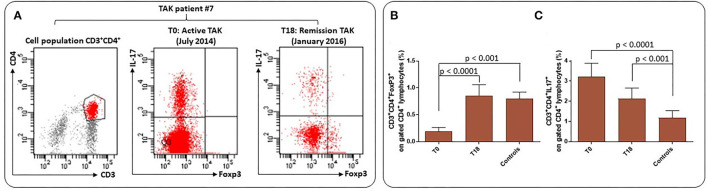
Frequency of Treg and Th17 cells in TAK patients and atherosclerotic Controls. **(A)** Flow cytometry analysis: gating was performed on live CD3^+^CD4^+^ cells to identify Foxp3+Treg and IL-17^+^ cells. Representative plots from one patient for each group. **(B)** Results are presented as mean ± S.D. of CD3^+^CD4^+^Foxp3^+^ percentages and CD3^+^CD4^+^IL-17^+^ T cells **(C)** in TAK patients before (T0) and after 18 months (T18) of infliximab treatment and in matched Controls (Wilcoxon signed-rank test and unpaired *t*-test, Mann-Whitney test).

In a subpopulation of sixteen TAK patients treated with infliximab, the frequency of CD4^+^FoxP3^+^Tregs and CD3^+^CD4^+^ interleukin (IL)-17^+^ cells were assessed before (time 0, T0) and after 18 months of treatment (time 18, T18) (Figure 4A). A subset of 15 Controls were included. Flow cytometry was performed on freshly isolated peripheral blood mononuclear cells (PBMCs) stimulated with phorbol 12-myristate 13-acetate (PMA) and ionomycin (calcium ionophore), activating protein kinase C, bypassing the T cell membrane receptor complex and leading to activation of several intracellular signaling pathways, resulting in T cell activation and production of a variety of cytokines (36). At T0, the mean percentage of Tregs was significantly reduced in TAK patients (0.22/0.67, *p* < 0.0001) compared to Controls and significantly increased at T18 (0.22/0.86, *p* < 0.001) (Figure 4B). At the same time, the frequency of CD3^+^CD4^+^IL-17^+^ cells indicating IL-17 expression behaved in the opposite way. The higher number of Th17 cells observed in TAK patients at T0 (3.2/1.4, *p* < 0.0001) significantly decreased at T18 (3.2/2.2, *p* < 0.001) (Figure 4C), likely as a result of infliximab treatment. Experiments performed to investigate function and/or viability of Tregs are, at this time, too preliminary to perform a statistical analysis.

Taken together, these results support the idea that biologic therapy may be useful to achieve better control of TAK and achieve a Treg/Th17 score similar to that of Controls. Further prospective and larger studies should be useful to understand whether these results may allow a possible decrease in steroid and immunosuppressant therapy and help to protect against cardiovascular risk.

## Discussion

This cross-sectional, single-center study aimed to investigate the potential association between parameters of vascular and cardiac impairment, infiltration of immune cells into vessel tissue, and overall risk of cardiovascular events in TAK patients. Patients with atherosclerosis, whose cardiovascular risk factors are well known, were selected as a control group.

The development of atherosclerosis is a recognized cardiovascular risk factor, and steroid therapies can have significant side effects on the lipid spectrum, with a notable impact on total and LDL cholesterol levels (37). Although steroid treatment is expected to prolong the life expectancy of TAK patients, it may contribute to an increased risk of cardiovascular events through dysregulation of plasma lipid levels. In our study, TAK patients had lower levels of total and LDL cholesterol compared with atherosclerotic patients, with no significant difference in HDL cholesterol or triglyceride levels between the two groups.

Due to the disease-related pathophysiological changes in vascular compliance, blood pressure levels are often elevated in TAK patients (38). However, while systolic blood pressure (SBP) values in TAK patients were similar to those in atherosclerotic patients, diastolic blood pressure (DBP) values in both left and right arms were significantly lower in TAK patients. As in Controls (38, 39), these results demonstrate that accelerated arterial stiffness and concomitant higher pulse wave velocity progression in our TAK patients may contribute to a significant increase in SBP and concomitant decrease in DBP values.

On echocardiographic ultrasound, the parameters of cardiovascular dysfunction in the TAK patients were comparable to those in the atherosclerotic patients: Both groups showed similar increases in LVM, IVS, and LAV. Similar to Controls (40, 41), concentric left ventricular hypertrophy is observed in TAK patients (14).

Mild-to-moderate and moderate-to-severe aortic regurgitation were observed in our TAK patients, with the most severe conditions correlating with the highest serum uric acid levels. Aortic regurgitation may be the result of aortic root dilatation, the risk of which increases in parallel with the rise in serum uric acid levels (42). According to recent epidemiological studies, elevated uric acid levels have been associated with the development of hypertension (42) and dyslipidemia (43), leading to a predictive marker for cardiovascular events (44). Uric acid enhances the transcription of nuclear factor-KB (45), which has pro-inflammatory and pro-atherogenic effects in the vascular wall (46), as it stimulates the production of inflammatory cytokines, including TNF-α (47), and the release of C-reactive protein (48). This protein in turn enhances the expression of cellular adhesion molecules, promotes cellular apoptosis, and leads to endothelial dysfunction and arterial stiffness (49). In addition, high uric acid levels have a pro-oxidant effect, triggering the oxidation of lipoproteins (50) while limiting the bioavailability of nitric oxide in the arterial wall (51–54). The decreased bioavailability of nitric oxide promotes endothelial dysfunction, increases vascular tone and consequently arterial stiffness (54, 55). Interestingly, in our TAK patients, diastolic dysfunction worsened with the increase in uric acid levels. The positive correlation found between increased ASI, uric acid levels and the degree of diastolic dysfunction in TAK patients explains the LV remodeling process in these patients.

LAVi was higher in TAK patients than in atherosclerotic patients. In addition to pressure and volume overload (56), LAV increases in conditions with systemic inflammation such as autoimmune diseases (13, 57) or pneumonia (58). The potential prognostic value of LAV dimensions for cardiovascular risk in TAK patients is interesting but deserves further investigation.

Carotid intima/media thickness (IMT), a surrogate marker of cardiovascular risk factors and atherosclerotic cardiovascular disease (ASCVD), was equally increased in both groups; in TAK patients, IMT was inversely correlated with aortic wall thickness. In addition, in TAK patients but not in Controls, common carotid IMT had a positive significant correlation to ASCVD. This suggests that inflammatory burden in TAK patients may represent an independent risk factor for cardiovascular or atherosclerosis-related disease, similar to canonical risk factors in atherosclerotic patients.

According to other reports (59–61), a significant decrease in total lymphocytes in peripheral blood and considerable infiltration of CD3^+^, CD4^+^, and CD8^+^ T cells in the arterial wall was observed in TAK patients during the active phase. The vessel wall also exhibited granulomatous infiltrates of CD4^+^ T cells and macrophages, neovascularization, loss of smooth muscle cells in the tunica media with damage to elastic membrane lamellae and elastin fibers, and growth of a lumen-constricting neointima, which may explain the increased ASI in these patients. Further evidence for these data is that ASI was positively correlated with total peripheral lymphocyte count and total CD3^+^ and CD3^+^CD4^+^ T-cell counts. While hypertension is associated with increased aortic stiffness independent of aortic wall thickness (21), we can therefore speculate that CD3^+^, CD4^+^, and CD8^+^ T-cell infiltrates and the resulting fibrotic outcomes are behind the increased ASI and aortic wall thickness in our TAK patients. To our knowledge, these findings described in TAK patients. Previous reports in giant cell arteritis did not show any correlation between peripheral T lymphocytes and arterial stiffness, evaluated with pulse wave velocity (PWV) (62). Conversely, in other vascular diseases, a role of T lymphocytes in vascular stiffness has been suggested by the relationship with increased PWV values (63).

Inflammation of vessel walls, mechanical or immune-mediated injury, and ischemia/reperfusion of tissues lead to the release of HMGB1 (High Mobility Group Box 1), a small nuclear protein that promotes DNA bending and preferential assembly of transcription factors at specific DNA domains (34, 64). When HMGB1 is secreted by activated immune cells into the extracellular environment, it acts on all cell populations involved in vascular inflammation and functions as a candidate signaling protein for the transition from acute inflammation to self-sustaining chronic inflammation in large vessel vasculitis (34, 64). Injured endothelial cells release HMGB1 (65) and attract endothelial cell progenitors that promote neovascularization (66). In addition, HMGB1 activates dendritic cells and promotes their functional maturation and responsiveness to chemokines and regulates cell migration (64). HMGB1 also attracts myocyte precursors and vessel-associated stem cells (67), which are required for intimal hyperplasia/angiogenesis typical of vessel remodeling in large vessel vasculitides (68). The significant increase in HMGB1 immunostaining in the arterial wall of our TAK patients and the direct correlation between HMGB1 and infiltration of CD15^+^ and CD4^+^ cells support the existence of a vicious circle that enhances autocrine/paracrine release of HMGB1 (25, 34, 65, 69) and alters vascular morphology and hemodynamic balance in TAK patients.

Compared with atherosclerotic patients, TAK patients had a high frequency of CD3^+^CD4^+^IL-17^+^ cells, indicating increased IL-17 expression, and lower levels of CD4^+^FoxP3^+^Treg cells in peripheral blood. Moreover, with respect to TAK patients receiving standard steroid therapy alone, infliximab co-treated patients showed significant improvement in clinical symptoms, allowing steroids to be discontinued more quickly. When these patients were assessed at T18 of combined therapy with infliximab, CD3^+^CD4^+^IL-17^+^, CD4^+^, and FoxP3^+^Treg cells substantially overlapped levels found in atherosclerotic patients. These results suggest that biologic therapy is highly effective compared with standard treatment in both improving clinical symptoms and controlling disease pathophysiology, as demonstrated by the more rapid de-escalation of daily prednisone dose and normalization of Tregs and Th17 cell frequency, and that infliximab facilitates immunologic balance in TAK patients as in atherosclerotic patients. Although these results are still preliminary and limited, they may serve as first evidence for future, statistically powered studies to examine the impact of biologic treatments on clinical outcomes in larger groups of TAK patients. In particular, it will be important to determine whether infliximab treatment can slow down, or even prevent the cardiovascular impairments associated with worsening of inflammatory status in TAK patients. This study has some limitations. Firstly, it was a single center study with small sample size, warranting future statistically powered studies in order to evaluate, in a proper prospective analysis, the impact on clinical outcome in terms of survival. All echocardiographic analyses could be significantly influenced by the quality of ultrasound images. Due to the disease studied, the hemodynamic state of right heart was evaluated only with ultrasound and no data on right heart catheterization were available. Additionally, all patients with comorbidities such as atrial fibrillation or lung disease were excluded, reducing the potential generalization of our results. Finally, the majority of TAK patients were under steroid treatment while Controls received anti-hypertensive treatment, and these two strategies may have different impact on vascular compliance. However, to our knowledge, there are no previous data analyzing cardiovascular risk in patients affected by TAK or the role of immune cells in cardiovascular risk in these patients.

## Conclusions

Overall, our results suggest that the severity of large vessel damage is higher in TAK patients than in Controls. The histological data suggest different pathogenic mechanisms underlying TAK or atherosclerosis. Interestingly, the increased ASCVD risk in TAK patients seems to be directly related to infiltration of the vessel wall by inflammatory cells, and damage-associated molecular patterns may play a key role in this mechanism. This may suggest that in systemic inflammation and vasculitis, optimal disease control may also reduce the residual risk of cardiovascular disease. As shown by our preliminary results on infliximab treatment, this drug seems to be particularly effective in restoring the normal frequency of Tregs and Th17 cells in TAK patients. On this basis, assessment of Tregs and Th17 populations could serve as a potential biomarker to monitor treatment efficacy and as a novel therapeutic target to reduce cardiovascular risk in TAK patients.

## Data Availability Statement

The raw data supporting the conclusions of this article will be made available by the authors, without undue reservation.

## Ethics Statement

The studies involving human participants were reviewed and approved by Ethics Committee of the University of Bari Medical School (Code number 06R76Y9-1). The patients/participants provided their written informed consent to participate in this study.

## Author Contributions

SC and VD contributed to the conception. SC, VD, CS, and GC contributed to design of the work. SC, VD, ACR, GC, SN, CS, AL, IS, and MP contributed to the acquisition of data. SC, VD, and AV contributed to data analysis and drafted the work. SC, VD, CS, AV, ACM, MF, and GI contributed to interpretation of data for the work. AS, GB, CC, LR, RR, and MM revised the manuscript critically for important intellectual content. All authors contributed to manuscript revision, read, and approved the submitted version.

## Funding

This work was supported by Programma Regionale—Research for Innovation REFIN—POR Puglia FESR-FSE 2014/2020; INNOLABS-POR Puglia FESR-FSE 2014-2020 (CITEL-Telemedicine Research Center) and Progetto Regione Puglia—Fondo europeo di sviluppo regionale e Fondo sociale europeo (FESR e FSE). The sponsors of this study are public or non-profit organizations that support science in general.

## Conflict of Interest

The authors declare that the research was conducted in the absence of any commercial or financial relationships that could be construed as a potential conflict of interest.

## Publisher's Note

All claims expressed in this article are solely those of the authors and do not necessarily represent those of their affiliated organizations, or those of the publisher, the editors and the reviewers. Any product that may be evaluated in this article, or claim that may be made by its manufacturer, is not guaranteed or endorsed by the publisher.
