# Supplementary material for: Cardiovascular Risk in Patients With Takayasu Arteritis Directly Correlates With Diastolic Dysfunction and Inflammatory Cell Infiltration in the Vessel Wall: A Clinical, ex vivo and in vitro Analysis
Source: Front Med (Lausanne). 2022 May 16;9:863150. doi: 10.3389/fmed.2022.863150 (PMC9149422; doi:10.3389/fmed.2022.863150)
Supplement: Supplementary file 1 [file Table_1.docx]

**Supplementary Table 1.** Different vascular involvement and activity findings in studied patients

| **Characteristic** | **TAK** | **Controls** | **p Value** |
| --- | --- | --- | --- |
| **Ascending Aorta** | 4 | 3 | Ns |
| **Thoracic Aorta** | 9 | 12 | Ns |
| **Abdominal Aorta** | 4 | 5 | Ns |
| **Carotid** | 22 | 26 | Ns |
| **Upper limbs arteries   (succlavia, axillary, brachial)** | 13 | 6 | Ns |
| **Iliac** | 4 | 6 | Ns |
| **Femoral** | 6 | 6 | Ns |
| **Lower limbs arteries** | 5 | 7 | Ns |
| **Mesenteric** | 2 | 1 | Ns |
| **Renal** | 2 | 1 | Ns |
| **Calcifications %** | - | 85% |  |
| **Vascular dilations %** | 12.5% | - |  |
| **Vessel wall neovascularization %** | 46.7% | - |  |
| **Vessel wall edema %** | 66.7% | - |  |

Ns = not significant.
